# Hypofractionated stereotactic radiotherapy for large or involving critical organs cerebral arteriovenous malformations

**DOI:** 10.2478/v10019-012-0046-7

**Published:** 2013-02-01

**Authors:** Sławomir Blamek, Dawid Larysz, Leszek Miszczyk, Adam Idasiak, Adam Rudnik, Rafał Tarnawski

**Affiliations:** 1 Radiotherapy Department, Maria Skłodowska-Curie Memorial Cancer Center and Institute of Oncology, Gliwice Branch, Gliwice, Poland; 2 Department of Neurosurgery, Medical University of Silesia, Katowice, Poland; 3 2^nd^ Clinic of Radiotherapy, Maria Skłodowska-Curie Memorial Cancer Center and Institute of Oncology, Gliwice Branch, Gliwice, Poland; 4 3^rd^ Clinic of Radiotherapy, Maria Skłodowska-Curie Memorial Cancer Center and Institute of Oncology, Gliwice Branch, Gliwice, Poland

**Keywords:** arteriovenous malformations, hemorrhage, hypofractionated stereotactic radiotherapy, obliteration, stereotactic radiosurgery

## Abstract

**Background:**

The treatment of large arteriovenous malformations (AVMs) or AVMs involving eloquent regions of the brain remains a challenge. For inoperable lesions, observation, volume-staged radiosurgery or hypofractionated stereotactic radiotherapy (HFSRT) are proposed. The aim of our study was to assess the safety and efficiency of HFSRT for large AVMs located in eloquent areas of the brain.

**Materials and methods:**

An analysis of records of 49 patients irradiated for cerebral AVMs with a mean dose of 19.9 Gy (12–28 Gy) delivered in 2–4 fractions with planned gap (at least one week) between fractions. Actuarial obliteration rates and annual bleeding hazard were calculated using Kaplan-Meier survival analysis and life tables.

**Results:**

Annual bleeding hazard rates were 4.5% and 1.6% after one and two years of the follow-up, respectively. Actuarial total obliteration rates were 7%, 11%, and 21% and total response rate (total and partial obliterations) 22%, 41%, and 55% after one, two and three years of the follow-up, respectively. There was a trend towards larger total obliteration rate in patients irradiated with fraction dose ≥ 8 Gy and total dose > 21 Gy for lesions of volume ≤ 8.18 cm^3^ which was not observed in case of partial obliterations.

**Conclusions:**

HFSRT results with relatively low obliteration rate but is not associated with a significant risk of permanent neurological deficits if both total and fraction doses are adjusted to size and location of the lesion. Predictive factors for total and partial obliterations can be different; this observation, however, is not firmly supported and requires further studies.

## Introduction

Treatment of large arteriovenous malformations (AVMs) is still a challenge for clinicians. The available options include surgery in rare cases, embolization, usually as a part of the combined treatment, stereotactic radiotherapy, and eventually, observation. Single dose radiosurgery in case of large-volume lesions is associated with a significant risk of morbidity.^1^,^2^ To reduce the risk of complications, volume-staged radiosurgery, repeated radiosurgery or hypofractionated stereotactic radiotherapy (HFSRT) are implemented. The reported results, however, are below expectation if compared with the results of stereotactic radiosurgery for selected, small and low-grade lesions. The most common system used for grading AVMs is the Spetzler-Martin grading system. It was originally developed for assessing the risk of surgery and the grade is the sum of points assigned for the size of the nidus ( <3 cm – 1, 3–6 cm – 2, >6 cm – 3), eloquence of the adjacent brain (non eloquent – 0, eloquent – 1), and type of venous drainage (superficial only – 0, deep – 1).^3^ If one thoroughly analyzes these reports, it can be easily seen that the best results of radiosurgery are reported in series with a great proportion of low-grade (Spetzler-Martin I and II) lesions, sometimes exceeding 60% of the whole group.^4^ When high-grade AVMs constitute a large proportion of the treated patients, the results are strikingly worse and for grade IV lesions obliterations of the order of 20% are reported.^1^ Some authors claim that large size of an AVM is not a risk factor for hemorrhage and in their series a higher risk of bleeding from smaller lesions was reported.^5^ On the other hand, there is an increasing evidence that large volume of an AVM can be an independent risk factor of bleeding after diagnosis, especially in case of high-grade AVMs with bleeding as a presenting syndrome.^6^,^7^ This observation is the rationale for stereotactic irradiation for otherwise untreatable large AVMs. The aim of our study was to evaluate the early outcome of patients treated with HFSRT for large AVMs or malformations involving structures prone to radiation damage like optic pathway or brainstem.

## Materials and methods

The analysis is based on records of 49 patients (25 women and 24 men) of age ranging between 14 and 71 years (mean 36), irradiated with HFSRT between 2003 and 2009. Mean volume of the lesion was 25.07 cm^3^, median 18.05 cm^3^ and ranged between 0.36 and 153.98 cm^3^. Most of the lesions were Spetzler-Martin grade III AVMs (36.7%), the detailed distribution of the lesions according to Spetzler-Martin grade is shown in Table 1.

The proportion of grade II AVMs in our series is quite significant. These patients were referred to radiosurgery by neurosurgeons because of comorbidities that increased the risk of anesthesia or surgery or because of the risk of severe complications, *e.g*. if the lesion was in an eloquent area or had both multiple deep draining veins and deep arterial feeders. Some of the patients preferred a non-invasive treatment and did not agree for neurosurgical intervention. Overall, all the patients were consulted by a neurosurgeon and either refused surgery or were referred to our department for treatment due to a high risk associated with the potential surgical intervention. Modified AVM score ranged between 0.95 and 16.11 with the mean and median value of 3.29 and 2.61, respectively. AVM score calculated according to the original formula was within the same range with mean and median values of 3.45 and 2.61, respectively. Eighteen (37%) patients presented with hemorrhage, 17 (34.7%) with epilepsy, 17 (34.7%) with paresis and 31 (63%) with headaches. At least one embolization before radiosurgery was performed in 28 (57%) (Table 2).

All radiosurgical procedures were performed using a linear accelerator (Clinac 2300 C/D, Varian Medical Systems, Palo Alto, USA) equipped with a micro-multileaf collimator (m3, BrainLab AG, Feldkirchen, Germany). Radiosurgery planning was based on the fusion of computed tomography (CT), magnetic resonance imaging (MRI) and magnetic resonance angiography (MRA) images. The target was defined as an AVM nidus without draining veins and outlined on the fused images. The PTV was not outlined separately but the leaves of the micro-multileaf collimator were moved 1–2 mm away from the target to account for positioning inaccuracy and possible intrafraction movement. The width of the margin was modified according to proximity of organs at risk. Thermoplastic masks were used for immobilization of the patients. To verify the position of the radiation isocenter the Winston-Lutz test was performed before irradiation. The treatment position was verified with electronic portal image device (EPID) generating MV images or with an on-board imaging device (OBI) acquiring orthogonal kV images used after the treatment unit upgrade to assure a better image quality precision of localization.^8^,^9^ If any shift was recorded, the treatment couch was moved and the imaging was made again to ensure the correct position of the patient. The patients were irradiated with 2–4 fractions of 6–11 Gy to total doses of 12–28 Gy. Mean and median total doses were 19.9 and 21 Gy, respectively. Mean and median fraction doses were 8.1 and 8 Gy, respectively. The most frequently used fractionation regimen was 3x7 Gy (14 patients) and 3x8 Gy (9 patients). The dose was prescribed according to lesion size and proximity of organs at risk and was specified at the isocenter. The dose constraint for the optic chiasm and optic nerves is 8 Gy for single fraction radiosurgery in our institution. The tolerable doses for hypofractionated regimens are not reliably defined. Consequently, we chose the fractionation scheme which allowed maintaining the dose for optic apparatus within 6–6.5 Gy per fraction in multifraction regimens. Several static conformal fields or fixed-angle intensity-modulated radiosurgery were used for irradiation whichever yielded a better dose distribution. The radiosurgical procedures are performed only once a week (Saturdays) in our center due to very high workload (linear accelerator used for radiosurgery operates on weekdays form 7 am. to 10 pm. and is used for conventional fractionated radiotherapy). Consequently, the overall treatment time varied according to the number of fractions and length of intervals between them. The mean and median overall treatment time was 7.8 and 6 weeks, respectively. Median and mean follow-up time was 23.8 and 28.9 months, respectively.

The investigators strictly followed recommendations of the Helsinki Declaration (1964, with later amendments) and of the European Council Convention on Protection of Human Rights in BioMedicine (Oviedo 1997).

The Kaplan-Meier analysis was used to determine actuarial obliteration rates. The annual bleeding hazard rate was calculated with Kaplan-Meier life tables. The log-rank test was used to compare outcomes of different groups defined according to selected patient- and treatment-related factors (sex, age, AVM volume, number of hemorrhages and embolizations, Spetzler-Martin grade, AVM score, fraction dose, total dose, number of fractions). All statistical evaluations were performed with Statistica 7.0 PL software.

## Results

**FIGURE 1.**
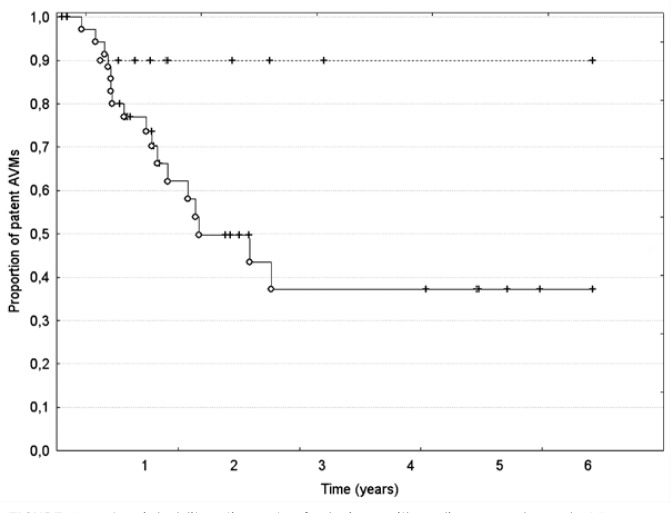
Actuarial obliteration rates for lesions with radiosurgery-based AVM score < 4 (solid line) and ≥ 4 (dashed line). The difference, although apparent, did not reach statistical significance (p = 0.06). O = complete; + = censored

Actuarial total obliteration (TO) rates after one, two, and three years of observation were 7, 11, and 21%, respectively. If partial obliterations (PO) were added, the cumulative response to the treatment (partial and total obliterations) was 22, 41, and 55%, respectively. According to Spetzler-Martin grade TO was seen in 4/15 (26.6%) grade II, 1/18 (5.5%) grade III, and 1/12 (8.3%) grade IV AVMs, whereas PO in 3/15 (20%), 6/18 (33.3%), and 4/12 (33.2%), respectively. None of grade V AVMs obliterated. Mean and median time to PO was 12.5 and 12.3 months, respectively, whereas mean and median time to TO was 16 and 12.8 months, respectively. The statistical analysis did not reveal significant differences in probability of obliteration (TO + PO) according to sex, age, Spetzler-Martin grade, number of fractions and hemorrhages. There was, however, a trend towards higher obliteration rates in patients with AVM score less than 4 (Figure 1).

**FIGURE 2.**
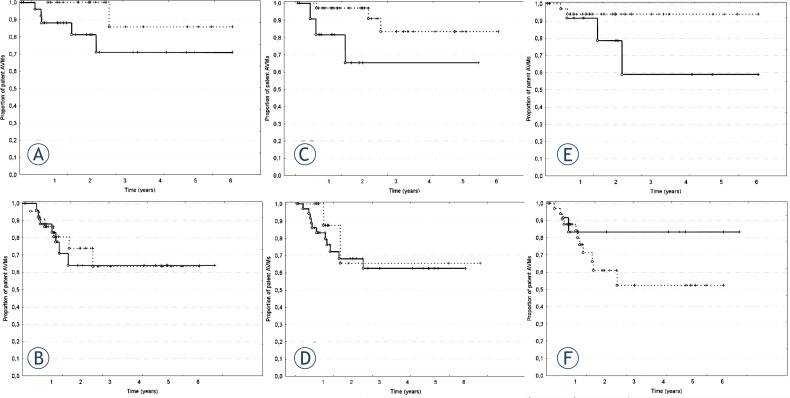
Actuarial obliteration rates for lesions treated with fraction dose ≥ 8 Gy (solid line) and < 8 Gy (dashed line); graphs A and B: A – complete obliterations; B – partial obliterations; total dose > 21 Gy (solid line) and ≤ 21 Gy (dashed line); graphs C and D: C – complete obliterations, D- partial obliterations; and of volume equal or smaller than 8.18 cm^3^ (solid line) and larger than 8.18 cm^3^ (dashed line); graphs E and F: E – complete obliterations, F- partial obliterations. O – complete; +- censored

Similar differences were observed when total obliterations were analyzed. AVMs irradiated with total dose >21 Gy, fraction dose ≥8 Gy, and of volume ≤ 8.18 cm^3^ which is an equivalent of a lesion of 2.5 cm diameter tended to have higher TO rates (p = 0.0518, p = 0.0937, and p = 0.0867, respectively, log-rank test). The differences, although apparent, did not reach the level of statistical significance. Interestingly, these factors did not show a tendency to influence the response rate in case of PO (p = 0.5895, p = 0.8357, and p = 0.2604, respectively, Figure 2).

In the group of partially obliterated AVMs the lesions that had previously been partially embolized tended to respond better than those treated *de novo* but this difference was also not statistically significant (p=0.0949). This trend, however, was not observed when TO were analyzed (p=0.3382).

Two patients bled after the treatment which resulted in annual bleeding hazard rate of 4.5 and 1.6% in the first and second year of the follow-up, respectively. Both hemorrhages occurred within the first four months after the treatment, before any treatment response could be seen. None of the patients bled before the completion of the treatment. The first patient with hemorrhage was a 40-years old male with a giant, grade IV AVM localized in the left thalamus and extending into the third ventricle. He had an episode of bleeding and one embolization before radiotherapy. He was treated with 18 Gy in two fractions. The other moved abroad and did not show-up for the scheduled follow-up visit. The information about bleeding was mailed from neurosurgery department taking care of the patient at that time. The patient was a 23-years-old female with a grade II AVM localized in the parasagittal, fronto-parietal region. She received 20 Gy in 2 fractions and was neither previously treated with endovascular methods nor had a hemorrhage before radiosurgery. The bleeding was diagnosed with CT imaging which revealed an intraventricular hemorrhage. Bilateral external ventricular drains were placed to relieve the symptoms of hydrocephalus. The patient finally recovered and was able to return to work. Apart from the AVM the patient had multiple aneurysms that could also have bled and because the source of bleeding was not clearly identified in the documentation we received, it should be in fact considered undetermined. If we assume that an aneurysm was the source of hemorrhage in this patient, the calculated annual hazard ratios for bleeding in our series would be 2.2 and 1.6% in the first and the second year of follow-up, respectively.

We observed transient neurological deterioration in six patients after the treatment. In most cases these were aggravated symptoms of paresis or epileptic seizures and headaches that resolved during the follow-up. In two patients an episode of sudden, severe headache and neurological deterioration occurred which was the reason for hospitalization and intensive diagnostic workup. In none of them a hemorrhage was diagnosed, in both, however, no blood flow through the AVM nidus was seen. In both patients the symptoms finally resolved to the level from before the treatment. No symptomatic necrosis after HFSRT was observed. We did not record any apparent signs of edema or demyelination asymmetric or shifted in relation to the target volume suggestive of geographical error. Consequently, we can assume that the position verification procedures were sufficient for dose delivery according to the treatment plan.

## Discussion

In our series the number of obliterated AVMs is low with only 21% of total obliterations and additional 34% of partial obliterations after three years of the follow-up. Nevertheless, if one takes into account very large median volume of the lesions treated, the results compare favorably with those presented in other papers. The obliteration rate depends mainly on the size and grade of the AVMs and also on the total and fraction dose applied.^1^,^10^,^11^ In publications showing the results of the treatment of exclusively high grade lesions the results are discouraging. Xiao *et al.* reported the results of the treatment with HFSRT a series of 20 AVMs greater than 5 cm. No lesion obliterated, nevertheless, they observed a significant decrease in the volume of patent AVM with residual volume ranging from 1.5–98% of initially treated lesion.^12^ The annual risk of bleeding after the treatment was 2.06% which is in line with our results and indicates that HFSRT does not increase the risk of subsequent hemorrhage.^12^ In a series of AVMs larger than 15 cm^3^ reported by Lee *et al.* they observed 25% of complete and 50% of partial obliterations after the mean follow-up of 41.2 months.^13^ In patients treated with proton beam radiosurgery for high-risk inoperable AVMs at the median follow-up of 56.1 months 15% of TO and 34% of PO were observed.^14^ Moreover, a significant actuarial rate of hemorrhage of 22% at 5 years was reported with large volume of the lesion being the risk factor for bleeding.^14^ Our data did not confirm the high risk of bleeding in the latency period. This may be the result of several factors like higher total doses applied in our series, shorter follow-up and patient selection. Some authors consider partially occluded AVMs more prone to bleeding and advocate a further treatment to reduce the risk of hemorrhage. This is certainly the case after partial embolization or partial resection of the lesion which causes sudden increase of blood pressure in the remaining and defective vessels of the nidus.^15^ Partial obliteration of an AVM after radiosurgery is a slower and gradual process. Histological changes in the vessels of an irradiated AVM make them thicker and more resistant to changes in blood pressure and thus, to rupture.^16^ There is also some evidence that in a long-term perspective, even partial treatment of an AVM can eventually be more advantageous than no treatment at all.^6^,^17^ Moreover, partially obliterated lesions can be further treated with stereotactic radiosurgery aimed at obliteration of the patent portion of the nidus or can become suitable for surgery.^12^,^18^,^19^

A relatively short follow-up can be considered a shortcoming of this paper. The increasing number of obliterations with extending follow-up time is well appreciated. We observed only 8% of TO after the 1^st^ year of the follow-up, whereas after 3 years it was already 21%. Lindvall *et al*. observed a raise in actuarial obliteration rates from 56% to 81% for lesions of volume 4–10 cm^3^ and from 50% to 70% for lesions larger than 10 cm^3^ after two and five years of the follow-up, respectively.^20^ Zabel du Bois *et al*. irradiated with HFSRT lesions of median volume of 27 cm^3^ which resulted in obliteration rate of 17% after three years. The proportion of obliterated lesions rose to 33% after four years of the follow-up.^10^ In the series reported by Chang *et al*. the 3-year, 5-year and 6-year actuarial obliteration rate for AVMs treated with HFSRT was 32, 61 and 71%, respectively.^21^ Taking into account much larger median volume of the AVMs in our series (18.5 cm^3^, whereas in their series only 28% AVMs had diameter >2.5 cm which results in volume of 8.18 cm^3^ assuming spherical shape of a lesion, the median volume was not given) the results after three years of the follow-up are comparable.^21^ Veznedaroglu *et al*. reported 22% obliteration rate after the 5-year follow-up in the group of patients irradiated with 30 Gy in 6 fractions. Irradiation with 7 Gy per fraction instead of 5 Gy resulted in total dose of 42 Gy and a high – 81% 5-year obliteration rate but at the cost of significant treatment-related morbidity. As a result, the dose of 7 Gy was considered too high to assure a safe treatment.^22^ We did not implement a fixed protocol of hypofractionation like in most other studies which assumed a fractionation schedule of 5–6 × 5 Gy, 5 × 6 Gy or 5 × 7 Gy irrespective of volume and localization of the lesion.^12^,^22^,^23^ Instead, we often used fraction doses higher than 7 Gy to maximize the biological effect of a single fraction. Nevertheless, along with the increase of the fraction dose we reduced the number of fractions to decrease the total dose and thus, the probability of complications. We expect, therefore, a higher 5-year total obliteration rate with 21% of total obliteration rate and another 34% of partial obliterations just after three years of the follow-up. On the other hand, Aoyama *et al*. reported a much higher three-year obliteration rate of 53% after HFSRT but with a higher total dose of 28 Gy given in 4 fractions. In their series however, 12/26 AVMs were grade I or II lesions and there was the same number of AVMs with maximum diameter <2.5 cm and >2.5 cm (mean 2.26 cm).^24^

An interesting observation is the difference between factors that may influence the total and partial obliteration rates. Although the level of statistical significance was not reached, there was a clear tendency towards higher probability of complete obliteration of AVMs of smaller size (≤2.5 cm) irradiated with fraction doses at least 8 Gy and total doses greater than 21 Gy which is in good agreement with other studies.^1^,^20^,^24^ Factors influencing a partial response for the treatment seem to be somewhat different. We observed a trend towards greater probability of PO only in patients who had at least one embolization before irradiation whereas the dose and size of the lesion seemed to play a lesser role. These observations, however, require further studies because of borderline statistical significance resulting from low number of obliterations and limited number of patients in our series.

Larger than usually reported intervals between fractions did not influence negatively the treatment results in any obvious way. The obliteration and bleeding rates appear slightly superior to those reported by the Boston group.^14^ We suppose that elongation of time between consecutive fractions can reduce the probability of complications. In our series we did not observe any serious treatment-related damage. The treatment schedule with elongated intervals between fractions is very scarcely represented in the literature. Irradiation with protons with at least one week interval between fractions was not associated with any complications that could be linked to the fractionation schedule. Moderate success of this treatment, like in our series, was rather associated with large volumes of the lesions and relatively low doses applied.^14^ Doses in the range reported in the current series proved to be safe and perhaps there is still some possibility to increase the total dose by adding fractions or increase dose per fraction.

The treatment of large AVMs still remains a challenge, especially in light of conflicting results of the treatment reported in the literature. Many clinicians advocate observation for patients with large AVMs following the results published by Han *et al*.^25^ In newer series, however, these results were not confirmed. Moreover, recent comprehensive analyzes suggest that large AVMs can be particularly prone to bleeding resulting with morbidity and mortality larger than usually reported for AVMs, especially if they presented with hemorrhage. Consequently, the active treatment is proposed in these patients to reduce the risk of subsequent bleeding with often debilitating or even fatal outcome.^6^

## Conclusions

HFSRT should be offered only to patients with AVMs unsuitable to surgery or single fraction procedure because of relatively low probability of obliteration after the fractionated irradiation. A low risk of hemorrhage after the treatment and a low rate of complications if the fraction and total dose are adjusted to the size and location of the lesion indicate that hypofractionated stereotactic radiotherapy can still be an attractive option for patients with otherwise untreatable AVMs. The probability of total and partial obliteration may be associated with distinct factors and possibly depend on more variables than radiation dose and size or volume of the lesion. Further studies are necessary to confirm these observations.

## Figures and Tables

**TABLE 1.** Distribution of Spetzler-Martin grades

**Grade**	**Number**	**Percent**
II	15	30.6
III	18	36.7
IV	12	24.5
V	4	8.2

**TABLE 2.** Number of embolizations before stereotactic radiotherapy

**Number of procedures**	**Patients**	**Percent**
None	21	43.6
1	9	18.7
2	5	10.4
3	7	14.5
4	3	6.2
5	2	4.1
6	1	2
